# Comparison of the Therapeutic Efficacy of Antibiotic-Loaded Polymeric Tissue Scaffold and Bone Cement in the Regeneration of Infected Bone Tissue

**DOI:** 10.7759/cureus.46487

**Published:** 2023-10-04

**Authors:** Petek Konya, Mehmet N Konya, Bilge Kagan Yilmaz, Elif Kaga, Sadık Kaga, Yeliz Çetinkol

**Affiliations:** 1 Infectious Diseases, Afyonkarahisar Health Science University, Afyonkarahisar, TUR; 2 Orthopeadics and Traumatology, Afyonkarahisar Health Science University, Afyonkarahisar, TUR; 3 Orthopaedics and Traumatology, Afyonkarahisar State Hospital, Afyonkarahisar, TUR; 4 Medical Services and Technique, Afyonkarahisar Health Science University, Afyonkarahisar, TUR; 5 Biomedical Engineering, Afyon Kocatepe University, Afyonkarahisar, TUR; 6 Medical Microbiology, Afyonkarahisar Health Science University, Afyonkarahisar, TUR

**Keywords:** osteomyelitis, daptomycin, ertapenem, bone cement, scaffold

## Abstract

Background

Local antibiotic applications have been used in chronic osteomyelitis and have been defined as an adjunctive treatment method. Biodegradable materials are also used for the same purpose by adding antibiotics. The fact that it does not require additional surgery to be removed is an important advantage. In this study, we intended to develop a new biodegradable drug-loaded polymeric scaffold with good antibiotic release and compare the microbiological results with antibiotic-impregnated bone cement.

Methodology

A tissue scaffold containing poly(2-hydroxyethyl methacrylate) (PHEMA) was prepared in our laboratory and loaded with ertapenem and daptomycin antibiotics. The surface morphology and pore geometries of drug-loaded and unloaded scaffolds were analyzed by a scanning electron microscope under vacuum. The dose-dependent antiproliferative effects of PHEMA scaffold, drug-loaded scaffold, cement, and drug-loaded cement on osteoblast cells were investigated. To evaluate drug release kinetics, the absorbance values of the scaffold loaded with ertapenem and daptomycin were measured with the spectrometer. For microbiological tests, ertapenem and daptomycin-impregnated cement and scaffold, as well as the control scaffold and cement samples, were investigated for their antibacterial activities on *Staphylococcus aureus* and *Klebsiella pneumoniae* strains using the disc diffusion method. These microorganisms are one of the most common microorganisms in osteomyelitis.

Results

The efficacy of antibiotic-impregnated scaffold and cement on both gram-negative and gram-positive microorganisms was investigated. The daptomycin zone diameter in *S. aureus *ATCC 29233 strain was 17 mm, whereas it was 24 mm for scaffold and 22 mm for cement. Scaffold was found to be more effective than cement against *S. aureus* strain. The *K. pneumoniae *ATCC BAA-2814 strain was found to be resistant to ertapenem, but the zone diameter was 21 mm for scaffold and 20 mm for cement. Ertapenem-loaded scaffold was found to be more effective than cement. It was found that the antimicrobial activity of the scaffold was higher than cement. When we evaluated the release profiles, for the daptomycin-loaded cement group, 98% of daptomycin was cumulatively released within 30 minutes, and for the daptomycin-loaded scaffold group, 100% of daptomycin was cumulatively released in six days. To compare ertapenem-loaded cement and scaffold, 98% of ertapenem was cumulatively released within 10 minutes in the cement group. For the scaffold group, 100% of ertapenem was cumulatively released in 17 days. We found that the scaffold released the antibiotic more slowly and for a longer duration. Therefore, it was thought that the scaffold would be more effective on biofilm and the treatment of osteomyelitis would be more successful.

Conclusions

The produced scaffold was compared with cement, and it was concluded that the scaffold had better release and antimicrobial efficacy. Scaffold is more advantageous than cement because it is bioeliminable. Thus, there is no need for a second surgical intervention with the likely prevention of mortality and morbidity. Because of all these features, the scaffold seems promising in the local treatment of osteomyelitis.

## Introduction

Osteomyelitis is defined as a progressive disease characterized by microorganisms causing damage to any part of bone tissue through infectious and inflammatory processes [1]. While osteomyelitis could follow a mortal course in the past, with improved surgical options, compliance with sterility, and antibiotic applications, it has been reduced to severe disease, and deaths have been almost completely prevented. However, despite the advances in today’s drug technologies and surgical treatments, it is difficult to reach the desired level of success [2].

The microorganisms causing osteomyelitis may vary depending on the age of the patient and the underlying diseases they have. *Staphylococcus aureus* stands out as the most frequently isolated pathogen in all types of osteomyelitis. Coagulase-negative staphylococci are frequently isolated in infections due to foreign bodies such as implants and prostheses [3]. In nosocomial infections and in elderly and immunosuppressed patients, *Pseudomonas aeruginosa*, *Klebsiella pneumoniae*, *Acinetobacter baumannii*, and *Escherichia coli* spp. are the responsible agents. In addition, streptococci, anaerobic bacteria, *Pasteurella multocida*, and *Eikenella corrodens* are frequently isolated from human and animal bites. Diabetic foot and decubitus ulcer osteomyelitis is a polymicrobial infection [4]. Moreover, *Bartonella henselae*, *Mycobacterium avium*, and fungi (*Aspergillus *species, *Candida albicans*) have been reported in immunocompromised patients [5].

According to Cierny-Mader, a two-stage treatment is applied in osteomyelitis surgery. The first stage involves radical debridement of dead bone and poorly vascularized soft tissues and systemic antibiotic therapy with placement of antibiotic-impregnated cement (AIC) (beads, rods, nails, or blocks) in the resulting bone defect. In the second stage, after six to eight weeks, the AIC is removed and the bone defect is filled with different methods [6]. AIC beads are used to sterilize and temporarily fill the dead cavity and are usually removed within two to four weeks [7]. The most commonly used antibiotics in these beads are cefazolin, moxalactam, cefotaxime, tobramycin, gentamicin, vancomycin, and ticarcillin [8]. The disadvantage of this method is the need for repeated surgeries such as bone grafting or bone lengthening surgeries to remove the cement and fill the bone defects. Due to these shortcomings, bioeliminable carrier systems have started to be developed. These methods aim to achieve the high local antimicrobial concentrations required in the treatment of chronic osteomyelitis while avoiding the need for surgical removal of the implant. In addition, bioeliminable materials eliminate dead cavities in the bone and accelerate the healing of local bone tissue [9].

Antibiotics used systemically in the treatment of osteomyelitis have limited efficacy because they do not penetrate well into cavities filled with dead bone and necrotic content. As these antibiotics are given in very high doses and for a long time for the same reasons, they have the disadvantages of side effects, systemic toxicity, long hospital stays, high cost, and the need for additional surgery [10]. For this reason, research on local carrier systems and local antibiotic applications is increasing day by day [11].

Besides being a biocompatible and non-toxic polymer, poly(2-hydroxyethyl methacrylate) (PHEMA) is a biomaterial used in many applications such as bone tissue regeneration, contact lenses, and wound dressings. In such applications, they are used in combination with cross-linkers such as ethylene glycol dimethylacrylate for network formation. Such chemical cross-linked network structures of PHEMA are not bioabsorbable [12].

Bioabsorbability is a property that depends on the molecular weight and molecular structure of the polymer. Bioabsorbable polymers are first broken down into short chains or monomers in the organism, and then these short chains and monomers are metabolized in the body or eliminated through the excretory system. Biocompatible polymers, on the other hand, when produced at the appropriate molecular weight (below the renal filtration threshold: 45 kDa), can be eliminated from the body [13]. Such structures are characterized as bioeliminable structures [14].

In this study, PHEMA-based and drug-loaded tissue scaffolds, which provide the advantage of bioelimination and slow drug release, were developed as an alternative to routinely used cross-linked polymethyl methacrylate (PMMA) structures. For this purpose, tissue scaffolds were produced with very small molecular weight (average Mn: 530 g/mol) PHEMA chains by solvent casting without the use of crosslinkers [15].

In this study, we aimed to evaluate the antimicrobial efficacy of antibiotics implanted in a PHEMA-based, bioeliminable, drug-loaded polymeric tissue scaffold with good antibiotic release, which can be an alternative to AIC.

This article was previously posted to the medRxiv preprint server on August 25, 2023.

## Materials and methods

Financial support for the study was received from the Afyonkarahisar University of Health Sciences Scientific Research Projects Unit with project number 22.GENEL.016.

Chemicals and equipment

Sodium metabisulfite was purchased from Acros organics, potassium persulfate from Akbel, and 2-hydroxyethyl methacrylate (HEMA) from TCI companies. Diethyl ether and ethanol used in the polymerization stage and tissue scaffold production and lyophilized ertapenem were supplied from the Merck company (Merck İlaç Ecza ve Kimya Ticaret AŞ, Istanbul, Turkey). Lyophilized daptomycin was supplied from Novartis (Novartis Sağlık, Gıda ve Tarım Ürünleri San. Tic. AŞ, Istanbul, Turkey). The surface morphology of the scaffolds was analyzed using LEO 1430 VP model scanning electron microscope (SEM) (Carl Zeiss AG, Jena, Germany) and BAL-TEC gold coating devices.

PHEMA synthesis

For the synthesis of PHEMA, a previously reported method was used [16]. First, 29 mg of potassium persulfate (0.1 mmol) and 29 mg of sodium metabisulfite (0.15 mmol) were dissolved homogeneously in 20 mL of 66.3% (v/v) ethanol/water mixture. Then, 5 g of HEMA monomer (38.4 mmol) was added and the reaction flask was sealed with a septum. Oxygen in the reaction medium was purged with high-purity nitrogen gas for 15 minutes. The reaction was continued on a magnetic stirrer at room temperature for six hours. The polymerization was then stopped by exposing the reaction to an open atmosphere. The polymer solution was dropped into 200 mL of cold ether and stirred on a magnetic stirrer in an Erlenmeyer to precipitate and purify PHEMA. The precipitated polymer was redissolved in 3 mL of ethanol and dropped into 200 mL of cold ether to dissolve again. This process was repeated once more and the polymer was purified by a total of three precipitations (Mn: 530 g/mol PDI: 1.2).

Tissue scaffold production

Tissue scaffolds were produced using the solvent-casting method. Both cementum and scaffold were impregnated with daptomycin and ertapenem as antibiotics. Four antibiotic-absorbed cements and scaffolds were used.

Ertapenem-Loaded Tissue Scaffolds

For this, 200 mg PHEMA and 50 mg ertapenem were dissolved in a 700 µL ethanol/water mixture (6:1) by vortexing. To produce disk-shaped tissue scaffolds for cell tests and microbiological tests, 50 µL of this mixture was dropped onto a glass surface and dried in an oven at 25°C for 24 hours.

Daptomycin-Loaded Tissue Scaffolds

For this, 200 mg PHEMA and 50 mg daptomycin were dissolved in a 700 µL ethanol/water mixture (6:1) by vortexing. To produce disk-shaped tissue scaffolds for cell tests and microbiological tests, 50 µL of this mixture was dropped onto a glass surface and dried in an oven at 25°C for 24 hours.

Drug-Free Tissue Scaffolds

For this, 250 mg PHEMA was dissolved in a 700 µL ethanol/water mixture (6:1) by vortexing. To produce disk-shaped tissue scaffolds for cell tests and microbiological tests, 50 µL of this mixture was dropped onto a glass surface and dried in an oven at 25°C for 24 hours.

SEM Analysis of the Tissue Scaffolds

The surface morphology and pore geometries of drug-loaded and unloaded scaffolds were analyzed by SEM under vacuum. After drying at 25°C for 24 hours, the samples were coated using a BAL-TEC surface coating device to increase the surface conductivity. The coating process was carried out by sputtering. The gold plating was performed under a 4 x 10^-2^ vacuum by applying 100 mA current for 20 seconds. The surface morphology of the scaffolds was then examined with a LEO 1430 VP model SEM device. Images were acquired in secondary electrons mode. Analysis was performed with an acceleration voltage of 20 kV.

Cell culture: proliferation test

A human osteoblast cell line (hFOB 1.19) was used in the study. Cells were incubated in 10% fetal bovine serum, 100 U/mL penicillin-streptomycin supplemented media at 37°C and 5% CO_2_. First, the dose-dependent antiproliferative effects of the antibacterial drugs ertapenem and daptomycin were tested. Cells (5 × 10^3^ cells/well) were seeded in 96 well plates the night before. The next day, the medium of the cells was replaced with a medium containing the drugs prepared at specific concentrations (0.01, 0.05, 0.5, 1.0, 3.0 mg/mL) and incubated for 48 hours under cell culture conditions. After incubation, cells were incubated with 0.5 mg/ml dimethylthiazol diphenyltetrazolium bromide (MTT) reagent in serum-free medium for four hours and then formazan crystals were dissolved with dimethyl sulfoxide. After treatment, absorbances were measured at 520 nm for each well using a plate reader (Thermo Scientific, Multiscan FC). At the same time, the antiproliferative effects of the scaffold, drug-loaded scaffold, cement, and drug-loaded cement on osteoblast cells were investigated. Cells (5 × 10^4^ cells/well) were seeded in 24 well plates and allowed to adhere for 24 hours. The next day, the scaffold, drug-loaded scaffold, cement, and drug-loaded cement were placed on the attached cells in the wells and incubated under culture conditions for 48 hours. After incubation, the MTT assay was performed as described above.

Release Kinetics

Daptomycin and ertapenem (20% (w/w)) were loaded onto polymeric tissue scaffolds to determine drug release kinetics. Each of the samples (10 mg) was then immersed in 10 mL of phosphate-buffered saline and shaken in an incubator shaker at 37°C. At certain time points (0 hours, 2 hours, 24 hours, 48 hours, 72 hours), absorbance values were measured by UV-VIS spectrometer. Measurements were performed by spectrophotometry (Shimadzu, uv-1280) using wavelengths of 364 nm and 300 nm for daptomycin and ertapenem, respectively.

Antimicrobial Tests

Two different tissue scaffolds (PHEMA and PMMA) to which ertapenem and daptomycin were loaded together with their control samples were sent to Afyonkarahisar University of Health Sciences Health Application and Research Center Microbiology Laboratory under sterile conditions. The antibacterial activity of the scaffolds on *S. aureus* ATCC 29213 and *K. pneumoniae* ATCC BAA-2814 strains was investigated by the disk diffusion method.

Preparation of the Bacterial Suspension at 0.5 McFarland (108 CFU/mL) Turbidity

A bacterial suspension was prepared in Mueller-Hinton broth (MHB) at 0.5 McFarland turbidity. For this purpose, one to two colonies of pure colony passaged bacteria were taken to prepare solutions at the 0.5 McFarland standard in a densitometer (BioMerieux, France).

Obtaining 10^6^ CFU/mL Concentrations of Bacteria by Serial Dilutions

For this, 1 mL of the original sample (10^8^ CFU/mL) was taken and added to 9 mL of MHB and mixed. The new sample had a cell concentration (cell count/mL) of 1/10 of the original sample. This process was repeated and serial dilutions were performed to obtain a bacterial concentration of 10^6^ CFU/mL. This step was performed separately for *S. aureus* ATCC 29213 and *K. pneumoniae* ATCC BAA-2814 strains.

Determination of Antimicrobial Activity of the Scaffolds by the Disk Diffusion Method

Daptomycin and ertapenem disks and the scaffold and cement controls were examined for their antimicrobial efficacy using the disk diffusion method. For this, 0.1 mL of the 10^6^ mL bacterial suspension was taken onto the blood agar and spread evenly over the entire surface of the medium using a pipette. Daptomycin and ertapenem were placed separately on each plate, and the daptomycin and ertapenem-loaded scaffolds and cements were also placed onto a plate using sterile forceps. The media were incubated in an oven at 37°C for 24 hours. One day later, the zone diameters on the plates removed from the oven were measured with a ruler and recorded.

## Results

Production of tissue scaffolds

**Figure 1:**
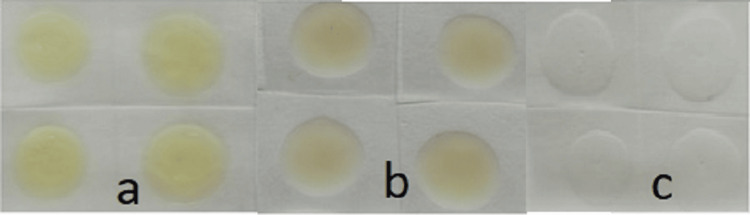
(a) Daptomycin-loaded scaffold, (b) ertapenem-loaded scaffold, and (c) free scaffold.

The dried tissue scaffolds prepared in the form of disks for cell culture and microbiological analyses are shown in Figure 1. Drug-loaded tissue scaffolds were prepared with 20% drug load and were macroscopically equivalent in size and appearance.

SEM analysis of the tissue scaffolds

**Figure 2:**
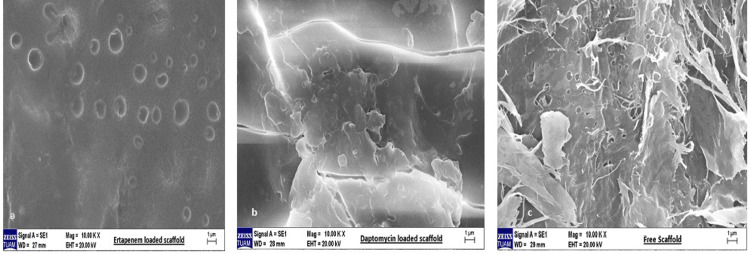
Scanning electron microscope images: (a) ertapenem-loaded scaffold, (b) daptomycin-loaded scaffold, and (c) drug-free scaffold.

SEM images of the ertapenem-loaded scaffold are shown in Figure 2a, the daptomycin-loaded scaffold in Figure 2b, and the drug-free scaffold in Figure 2c.

When the surface morphology of the scaffolds was examined, each had a different surface morphology. The drug-loaded scaffolds showed a more integrated structure than the drug-free scaffolds, whereas the drug-free scaffolds had a surface morphology with fibrillar extensions with more surface area.

Cell proliferation assay

**Figure 3:**
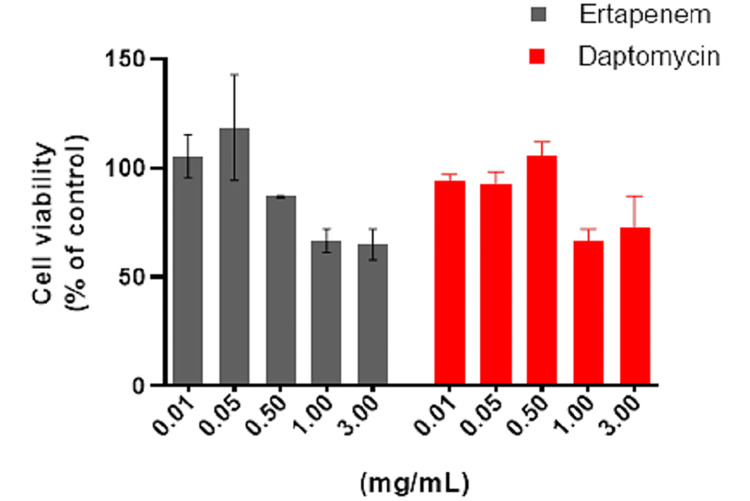
Effects of ertapenem and daptomycin on cell viability measured by the dimethylthiazol diphenyltetrazolium bromide assay. Osteoblast cells were treated with ertapenem and daptomycin at various concentrations (0.01-3 mg/mL) for 48 hours. Results are represented as the mean ± standard deviation of triplicate independent experiments.

The antiproliferative effects of ertapenem and daptomycin on osteoblast cells were examined. The drugs showed a dose-dependent antiproliferative effect. When both drugs were applied at doses of 0.01 mg/mL, 0.05 mg/mL, and 0.5 mg/mL, cell viability rates were 90%, whereas the cells where the drugs were applied at increasing doses (1 mg/mL and 3 mg/mL) had a rate of cell death of approximately 40% (Figure 3).

**Figure 4:**
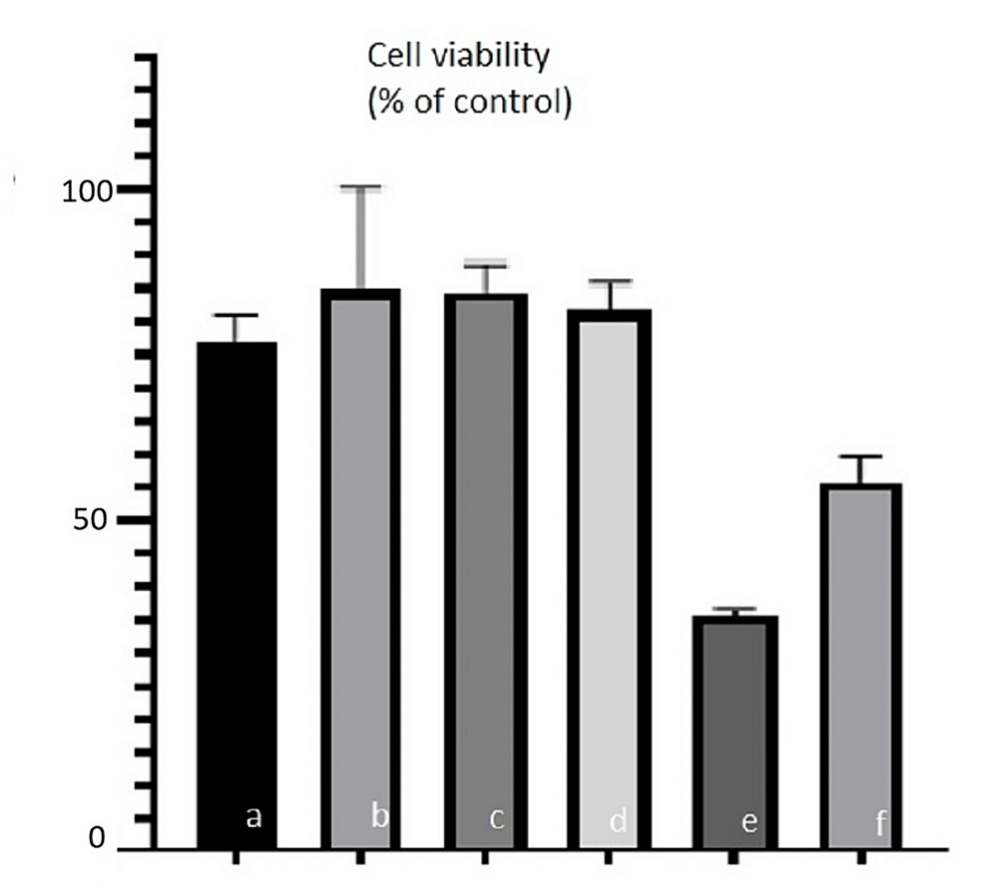
MTT assay for osteoblast cell proliferation on (a) polymeric scaffold, (b) cement, (c) ertapenem-loaded scaffold, (d) ertapenem-loaded cement, (e) daptomycin-loaded scaffold, and (f) daptomycin-loaded cement. Bar express mean ± standard deviation of triplicate independent experiments.

The antiproliferative effect of ertapenem and daptomycin-loaded scaffold and cement on osteoblast cells was investigated. Approximately 90% of cell viability was detected in the ertapenem-loaded scaffold and ertapenem-loaded cement group. In the daptomycin-loaded scaffold and daptomycin-loaded cement group, cell viability was approximately 50% (Figure 4).

**Figure 5:**
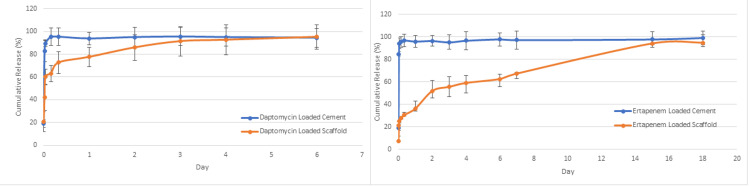
Release profile of (a) daptomycin and (b) ertapenem on a polymeric scaffold. 20% (w/w) daptomycin and entarpenem were loaded onto each scaffold, and the amount of released drugs was quantified by spectrophotometry. Error bars indicate standard deviation, n = 3.

Daptomycin and ertapenem were loaded onto both scaffold and cement to demonstrate the comparative release profile of scaffold and cement. In the daptomycin-loaded cement group, approximately 98% of daptomycin was cumulatively released within 30 minutes. In the daptomycin-loaded scaffold group, approximately 100% of daptomycin was cumulatively released in six days (Figure 5a). When ertapenem-loaded cement and ertapenem-loaded scaffold were compared, approximately 98% of ertapenem was cumulatively released within 10 minutes in the ertapenem-loaded cement group. In the ertapenem-loaded scaffold group, approximately 100% of ertapenem was cumulatively released in 17 days (Figure 5b).

Microbiological results

**Figure 6:**
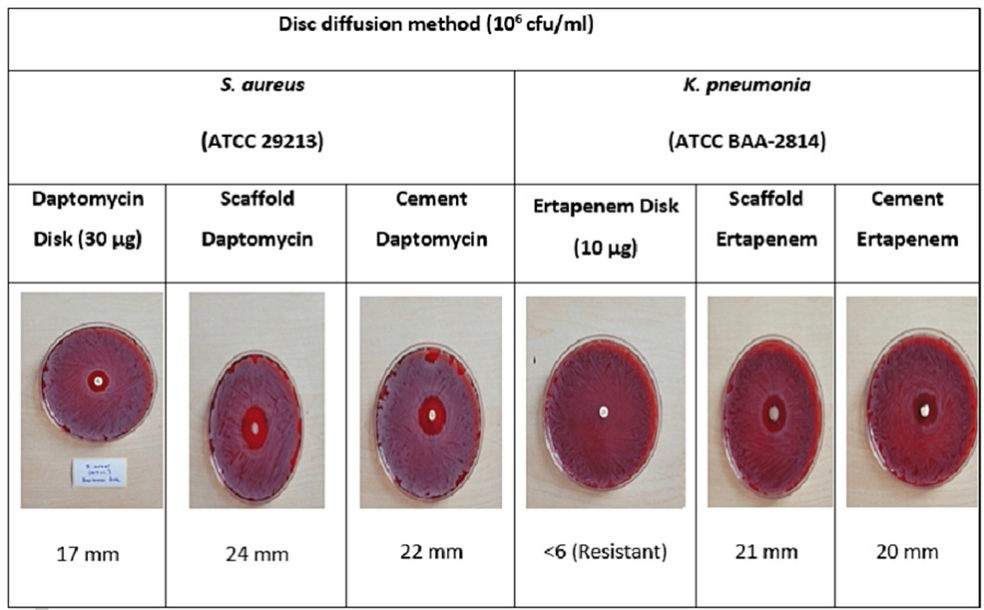
Comparison of the efficacies of scaffold and cement with those of antibiotic discs according to the disk diffusion method.

The efficacy of antibiotic-loaded scaffold and cement on the two most common microorganisms was investigated using the disk diffusion method (Figure 6).

In the *S. aureus* ATCC 29213 strain, the daptomycin zone diameter was 17 mm, whereas this value was 24 mm for scaffold and 22 mm for cement. Scaffold was found to be more effective than cement against *S. aureus* ATCC 29213 strain, whereas both building materials were more effective than daptomycin disk.

When the *K. pneumoniae* ATCC strain was analyzed, this strain was found to be resistant to ertapenem, but the zone diameter was 21 mm for the scaffold and 20 mm for the cement. Of the ertapenem-impregnated building materials, the scaffold was found to be more effective than bone cement, and both scaffold and bone cement were found to be more effective than the ertapenem disk.

As a result, the antimicrobial efficacy of the scaffold was found to be better than cement according to the disk diffusion method.

## Discussion

Today, PMMA cement is considered the gold standard antibiotic carrier for the treatment of osteomyelitis [17]. The main advantage of local antibiotic application with cementation is the high local antibiotic concentrations achieved, minimizing the risk of systemic toxicity and preventing emerging antibiotic resistance. Keeling et al. experimentally demonstrated that the bacterial biofilm layer can be reduced with vancomycin and daptomycin-impregnated cement in their animal study [18]. In a study by Bor et al. on 16 patients with chronic osteomyelitis, radical debridement was performed and AIC was applied to the bone defect. No recurrence was detected during a mean follow-up period of six years. However, in this study, the removal of cement with an additional operation is considered a disadvantage [19]. Therefore, in recent years, the development of bioeliminable tissue scaffolds that can be used instead of cement has gained momentum. With bioeliminable scaffolds, it is aimed to utilize both the local release effect of antibiotic-loaded scaffolds and avoid the need for a second surgery. Sambri et al. developed a bioeliminable scaffold consisting of a combination of calcium phosphate and nanocrystalline hydroxyapatite and investigated its local effect by impregnating the scaffold with antibiotics in animal models of osteomyelitis. In this study with single-stage debridement, favorable results were obtained despite the small number of subjects and a short follow-up period [20]. Alegrete et al. used a ceramic biomaterial loaded with antibiotics in the treatment of osteomyelitis in animal models. The ceramic biomaterial used was found to be effective in the treatment of osteomyelitis while supporting bone formation and osteointegration [21].

In this study, we produced a bioeliminable tissue scaffold containing PHEMA as an alternative to AIC. We loaded ertapenem and daptomycin on the produced scaffold and evaluated the release kinetics and antimicrobial activity in an in vitro environment.

The selection of the right antibiotic loaded onto the scaffolds, the way it is combined with cement, the amount of antibiotic, the release time, and the form of the drug in the surrounding tissues are of great importance in its use. The selected antibiotic should contain a broad antibacterial spectrum, including water-soluble, non-allergenic, and thermostable [22]. The most commonly used antibiotics are cefazolin, moxalactam, cefotaxime, tobramycin, gentamycin, vancomycin, and ticarcillin [8]. The rate of methicillin resistance in *S. aureus*, the most common causative agent of osteomyelitis, is increasing day by day. In a study by Ahmed et al. among patients with soft tissue infections, methicillin-resistant *S. aureus* (MRSA) was detected in 89.9% of the *S. aureus* isolates [23]. Hence, the treatment options MRSA effective antibiotics should be considered. In our study, we added daptomycin, which may be effective against possible gram-positive agents, into the scaffold we produced. Daptomycin is an antibiotic with bactericidal activity against gram-positive agents with MRSA activity [24]. *K. pneumoniae* is one of the most frequently isolated gram-negative agents in cases of osteomyelitis. In studies conducted on osteomyelitis, cephalosporin resistance is around 80% in *K. pneumonia* strains [25]. Carbapenems remain a good treatment option for the *Enterobacteriaceae *family that produces extended-spectrum beta-lactamase. In our study, we added ertapenem, an effective carbapenem against possible gram-negative agents, into the scaffold we produced.

In the literature, studies have been conducted on the antimicrobial activity of many tissue scaffolds on these microorganisms. Campos et al. compared vancomycin-loaded cement and calcium sulfate-containing tissue scaffolds on *S. aureus* strains. This study showed that both calcium sulfate-containing tissue scaffolds and cement release antibiotics for up to two weeks and act as good antibiotic carriers. However, scaffolds containing calcium sulfate showed greater antimicrobial release potential than cement. Therefore, it was concluded that tissue scaffolds containing antimicrobial-impregnated calcium sulfate showed high bioactivity to kill biofilm cells. In this study, the efficacy on gram-negative resistant agents was not evaluated, which is a limitation of the study [26].

In our study, the efficacy of antibiotic-impregnated scaffold and cement on both gram-negative and gram-positive microorganisms was investigated using the disk diffusion method. The daptomycin zone diameter in *S. aureus* ATCC strain was 17 mm, whereas this value was 24 mm for scaffold and 22 mm for cement. Scaffold was found to be more effective than cement against *S. aureus* ATCC strain, whereas both building materials were more effective than the daptomycin disk.

The *K. pneumoniae* ATCC strain was found to be resistant to ertapenem, but the zone diameter was 21 mm for scaffold and 20 mm for cement. As a result, the ertapenem-loaded scaffold was more effective than cement and both cement and ertapenem-loaded scaffold were more effective than the ertapenem disk. Accordingly, we found that the antimicrobial efficacy of the scaffold we developed was better than cement. Therefore, we think that a scaffold is a good alternative to cement.

We also evaluated the controlled release profile of the scaffold loaded with daptomycin and ertapenem in comparison with the release profile of cement, the most commonly used antibiotic carrier in daily practice. In the daptomycin-loaded cement group, approximately 98% of daptomycin was cumulatively released within 30 minutes. In the daptomycin-loaded scaffold group, approximately 100% of daptomycin was cumulatively released in six days. To compare ertapenem-loaded cement and scaffold, approximately 98% of ertapenem was cumulatively released within 10 minutes in the ertapenem-loaded cement group. For the ertapenem-loaded scaffold group, approximately 100% of ertapenem was cumulatively released in 17 days. As a result, we found that the scaffold we developed released the antibiotic more slowly and for a longer duration. It is thought that a bioeliminable scaffold that offers controlled and slow drug release may be more effective on biofilm and more successful in the treatment of osteomyelitis [27].

There are some limitations of this study. In this experimental study, we applied only two bacterial strains, which are the most common gram-positive and gram-negative strains, and only two antibiotics. This is a new structure and there are no in vitro and clinical studies on this subject. Further clinical studies are necessary and should be performed.

## Conclusions

In our study, the scaffold we produced was compared with cement, which we frequently use in daily practice. The produced scaffold had better drug release and antimicrobial efficacy. In addition, our scaffold is more advantageous than cement because it is bioeliminable. Thus, a second surgical intervention will not be necessary, and possibly mortality and morbidity will be prevented. Because of all these features, the scaffold seems promising in the local treatment of osteomyelitis. Our study was conducted under in vitro conditions and further extensive studies with animal experiments are needed.
